# Led astray by 16S rRNA: phylogenomics reaffirms the monophyly of *Methylobacterium* and lack of support for *Methylorubrum* as a genus

**DOI:** 10.1093/ismejo/wraf011

**Published:** 2025-01-21

**Authors:** Alexander B Alleman, Sergey Stolyar, Christopher J Marx, Jean-Baptiste Leducq

**Affiliations:** Department of Biological Sciences, University of Idaho, 875 Perimeter Drive MS 3051, Moscow ID 83844-3051, United States; Department of Chemical Engineering, University of Washington, 3781 Okanogan Lane, Seattle WA 98195-1750, United States; Department of Biological Sciences, University of Idaho, 875 Perimeter Drive MS 3051, Moscow ID 83844-3051, United States; Département de Phytologie (FSAA), IBIS, CRIV, 2480 Bd Hochelaga, Université Laval Québec, QC G1V0A6, Canada

**Keywords:** *Methylobacterium*, *Methylorubrum*, phylogeny, genomics, metagenomics, 16S rRNA gene, C_1_ metabolism, phyllosphere

## Abstract

Although the 16S (and 18S) rRNA gene has been an essential tool in classifying prokaryotes, using a single locus to revise bacteria taxonomy can introduce unwanted artifacts. There was a recent proposition to split the *Methylobacterium* genus, which contains diverse plant-associated strains and is important for agriculture and biotechnology, into two genera. Resting strongly on the phylogeny of 16S rRNA, 11 species of *Methylobacterium* were transferred to a newly proposed genus *Methylorubrum*. Numerous recent studies have independently questioned *Methylorubrum* as a valid genus, but the prior revision has left discrepancies among taxonomic databases. Here, we review phylogenomic and phenotypic evidence against *Methylorubrum* as a genus and call for its abandonment. Because *Methylobacterium sensu lato* forms a consistent and monophyletic genus, we argue for the restoration of the former and consensual *Methylobacterium* taxonomy. The large genomic, phenotypic, and ecological diversity within *Methylobacterium* however suggests complex evolutionary and adaptive processes and support the description of the most basal clade of *Methylobacterium* (group C) as a distinct genus in future work. Overall, this perspective demonstrates the danger of solely relying upon the 16S rRNA gene as a delimiter of genus level taxonomy and that further attempts must include more robust phenotypic and phylogenomic criteria.

## Introduction

*Methylobacterium* (*Methylobacteriaceae*, *Hyphomicrobiales*, *Alphaproteobacteria*; type species: *Methylobacterium organophilum* [1]) is a genus of Gram-negative, rod-shaped bacteria able to metabolize single-carbon compounds like methanol. Because these bacteria are often pink or reddish due to carotenoid pigments, they are referred to as pink-pigmented facultative methylotrophs. *Methylobacterium* species are primarily free-living and commonly found in various environments, including soil, water, and particularly in association with plants. Some *Methylobacterium*, like *Methylobacterium nodulans*, can form root nodules, promoting nitrogen fixation and uptake by plants [2], whereas others across the genus are found on the leaf surface (phyllosphere), establishing beneficial interactions with their host [3]. In the phyllosphere*, Methylobacterium* uses methanol as a primary carbon source, which is produced by plants as a byproduct of pectin metabolism and emitted through stomata [4]. They benefit the host by preventing pathogens, promoting growth through the production of phytohormones, and aiding in nutrient uptake [5–7]. The ability of *Methylobacterium* to utilize methanol and its benefits to plants makes it a model for biotechnology, already used commercially for protein production, bioremediation, and as a biostimulant in agriculture [3, 8, 9].

In 2018, Green and Ardley [10] proposed a significant taxonomic revision within the genus *Methylobacterium*, based on 16S rRNA gene sequences, multi-locus sequence analysis, variation in methylotrophic capabilities, and reddish pigmentation. The authors suggested that 11 species of *Methylobacterium* form a genetically and phenotypically homogeneous clade, and classified them into a new genus called *Methylorubrum*. Notable reclassified species included *Methylobacterium extorquens*, the best-studied methylotroph and the species in the genus most used in biotechnology, which was subsequently proposed as the type species of *Methylorubrum*.

Several recent phylogenomic-based studies have raised doubts about the genetic distinctions used to separate *Methylorubrum* from *Methylobacterium*. Hördt *et al.* found that although *Methylorubrum* is monophyletic, it causes *Methylobacterium* to be paraphyletic, violating key taxonomic guidelines [11]. Alessa *et al.* also argued against classifying *Methylorubrum* as a separate genus [12] and more recently, we supported this perspective, concluding that the genetic differences between the two groups are minor and should be viewed as variations within a single genus rather than justifying a taxonomic split [13]. Despite those controversies, both *Methylobacterium* and *Methylorubrum* are officially adopted as genera in major taxonomic databases, such as the List of Prokaryotic names with Standing in Nomenclature, the National Center for Biotechnology Information, and the most recent release of SILVA (v. 138) database for 16S rRNA gene microbial sequences [14], creating great inconsistencies among microbial studies.

Here, we examine recent phylogenomic and phenotypic studies that challenged the classification of *Methylorubrum* as a distinct genus and advocate for its abandonment and the reinstatement of the original and widely accepted *Methylobacterium* taxonomy (Patt *et al.* 1976). We argue that *Methylobacterium sensu lato* (Patt *et al.* 1976) represents a cohesive, monophyletic genus. We acknowledge that the significant phenotypic and genetic diversity within *Methylobacterium sensu lato* is however larger than expected for a genus and indicates complex evolutionary and adaptive processes, warranting further genomic, phenotypic, and ecological research on this group.

## Phylogenomic evidence against *Methylorubrum*

**Figure 1:**
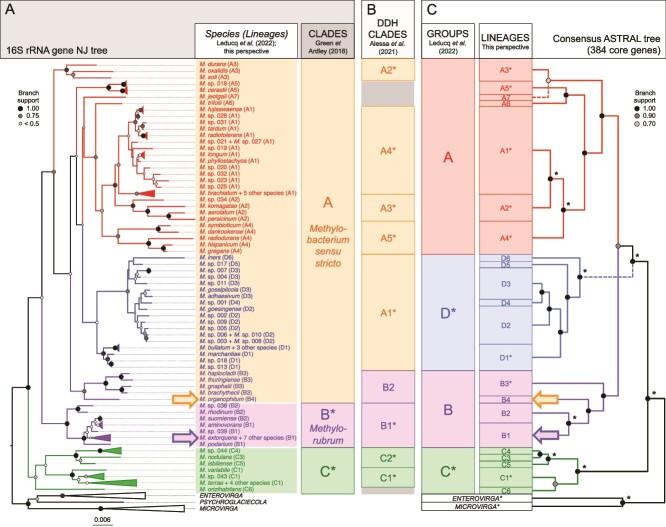
Comparison of recent *Methylobacterium* classifications showing that the 16S rRNA gene is a reliable taxonomic marker to distinguish most species but is inadequate to infer evolutionary relationships among them. (A) Clades based on the 16S rRNA gene tree [10]. Phylogeny was performed de novo from the complete nucleotide sequence of the 16S rRNA gene (neighbor-joining tree; 1000 replicates; sequences extracted from genomes previously used [13], but for *Psychroglaciecola*). Scale represents substitutions per site. (B) Clades based on DNA–DNA hybridization from complete genomes of 59 species (DDH clades; [12]). (C) Groups based on the consensus phylogeny from core genome (right; 384 core gene phylogenies combined with ASTRAL-III, simplified from previously [13]; only topology is showed; see Fig. 2A) and refined in well-supported lineages (this perspective). Arrows indicate type species *M. organophilum* and *Methylorubrum extorquens*. Clades, groups, lineages, and nodes in the consensus tree that are also monophyletic in the 16S rRNA gene tree are indicated by asterisks. Trees were arranged to minimize crossing branches (dotted lines in the consensus tree). In the 16S rRNA gene tree, species that could not be distinguished were collapsed together. Lineage C2 is missing (no complete 16S rRNA gene sequence available for *M. Crusticola*).

The original motivation for the *Methylorubrum* description by Green and Ardley [10] was to solve the large genetic diversity observed at the 16S rRNA gene within *Methylobacterium*—larger than what was expected for a single genus—as well as the fact that *M. organophilum*, originally chosen as the type species of the genus by Patt *et al.* [1], was much less studied than its famous counterpart, *M. extorquens.* Based on a phylogeny of the complete 16S rRNA gene sequence, the authors noted that *Methylobacterium* consisted of three monophyletic clades: A, B, and C. For the purpose of this perspective, we performed de novo a phylogenetic analysis using complete 16S rRNA gene nucleotide sequences found across 213 *Methylobacteriaceae* genomes from our previous study [13] and recovered the three clades described by Green and Ardley (Fig. 1A)**.** Clade A included the type species *M. organophilum* and, accordingly, was retained in *Methylobacterium* by Green and Ardley (*Methylobacterium sensu stricto*). Clade B was renamed as *Methylorubrum* and included the model species *M. extorquens*, which was naturally chosen by Green and Ardley as the type species. Finally, the basal clade C was retained in *Methylobacterium*, pending further taxonomic revisions.

At this point, it is worth noting several issues raised by phylogenetic analyses used by the authors to support *Methylobacterium* (Patt et al., 1976) emendation in *Methylobacterium* (clades A + C) and *Methylorubrum* (clade B). First, it is surprising that, given its clear genetic distinction and basal position, Green and Ardley did not propose an emendation for group C, as they did for group B, probably because it did not include enough genetic data or a model species like *M. extorquens*, or represented too much phenotypic complexity in comparison to its apparent genomic homogeneity. Alternative gene phylogenies explored by Green and Ardley (housekeeping, ribosomal, methylotrophy, and serine cycle genes), although confirming that clade B (*Methylorubrum*) was consistently monophyletic, also highlighted C as the most basal clade, making A + C paraphyletic. Second, Clade A, in addition to being poorly supported in the 16S rRNA gene phylogeny (<70%), was also poorly supported—or even paraphyletic—in alternative gene phylogenies presented by Green and Ardley. Finally, in the two alternative gene phylogenies in which *M. organophilum* was included by the authors (*gyrB* and *mxaF*), the *Methylobacterium* type species did not branch with clade A but formed a well-supported monophyletic group with clade B (*Methylorubrum*). Despite these profound discrepancies between the 16S rRNA gene phylogeny and alternative gene phylogenies, these were not addressed by the authors. Eventually, *Methylorubrum* was progressively adopted as a valid genus name in taxonomic databases.

**Figure 2:**
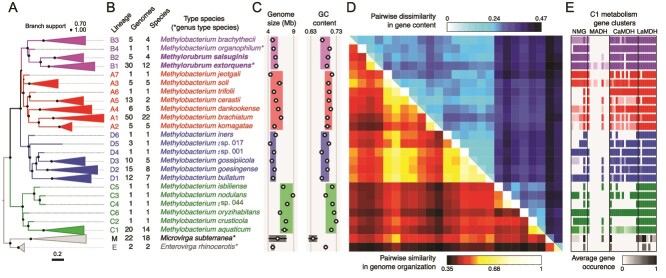
Genome characteristic support the definition of four monophyletic *Methylobacterium* groups. Data from 213 *Methylobacteriaceae* genomes [13]. (A) Consensus phylogeny from core genome (right; 384 core gene phylogenies combined with ASTRAL-III) collapsed for well-supported lineages and rooted on *microvirga* and *Enterovirga*. Scale represents coalescent units. (B) Lineage names, number of genomes and species, and type species per lineage. (C) Genome size and GC content (mean and standard deviation per lineage). (D) Pairwise dissimilarity in gene content (above diagonal) and pairwise similarity in genome organization (below diagonal). (E) Average gene occurrence per lineage for four methylotrophic metabolic pathways: NMG (8 genes encoding the N-methylglutamate pathway for methylamine utilization); MADH: (11 genes encoding for methylamine dehydrogenase); CaMDH: (14 genes encoding for the calcium-dependent methanol dehydrogenase) and LaMDH (6 genes encoding for the lanthanide-dependent methanol dehydrogenase).

Two studies have since questioned whether the genetic differences used to justify the separation of *Methylorubrum* from *Methylobacterium* are significant enough to warrant a distinct genus. By analyzing the genome of 62 type strains from *Methylobacterium* and *Methylorubrum*, Alessa *et al.* redefined clades on the basis of whole-genome-based DNA–DNA Hybridization (DDH) [12]. Authors showed that *Methylorubrum* was likely nested within Green and Ardley’s clade A, and that *M. organophilum*, the type species of *Methylobacterium*, and other relatives (e.g. *M. brachythecii*; clade B2 in Alessa *et al.* study; see Fig. 1B), shared more genomic similarity with *Methylorubrum* than with other *Methylobacterium* species from clade A. Accordingly, authors amended *Methylobacterium* back to the Patt *et al.* (1976) description, but the emendation was not fully adopted by the community [12]. Similarly, we recently reconstructed the consensus evolutionary tree of *Methylobacteriaceae* using 384 core genes and demonstrated that *Methylobacterium*-*Methylorubrum* consisted of four well-supported monophyletic groups (A, B, D, C; Figs 1C and 2A) [13], broadly consistent with DDH-based clades (Fig. 1B) [12].

We summarized the characteristics of 213 *Methylobacteriaceae* genomes we previously examined: genome size, GC content, gene content, and core genome architecture (synteny) (Fig. 2A–D) [13]. For this study, we also calculated Average Nucleotide Identity (ANI) among those genomes (Fig. S1). Although a 74% threshold is traditionally used to delineate bacteria genera with this statistic [15], *Methylobacterium sensu lato* formed a cohesive group for ANI = 80% and could not be distinguished from its sister genera for ANI = 79%, stressing the previously noted need to modify the criterion for genus delineation in *Hyphomicrobiales* [16]. All of the genome characteristics examined show both that (1) Group C can be clearly distinguished from the rest of *Methylobacterium* and that (2) *Methylorubrum* (lineages B2 and B1) does not represent a natural group (Fig. 2A–D; S1). Group C corresponded to Green and Ardley’s clade C (*Methylobacterium aquaticum, M. nodulans,* and relatives); its basal position remained unchanged compared to the 16S rRNA gene phylogeny, suggesting that it could be described *a fortiori* as a distinct genus, provided that we consider a highly conservative ANI threshold (82%; Fig. S1). Group B contained *Methylorubrum*, as well as some species from Green and Ardley’s clade A, including the *Methylobacterium* type species *M. organophilum*. The co-occurrence of two type species, *M. organophilum* and *Methylorubrum extorquens*, in this same group is in violation of taxonomic rules and invalidates *de facto* the previous emendation of *Methylobacterium* by Green and Ardley. The rest of the species from Green and Ardley’s clade A could be divided in two distinct groups: A (*Methylobacterium brachiatum and* relatives) and D (*Methylobacterium gossipiicola* and relatives).

**Figure 3:**
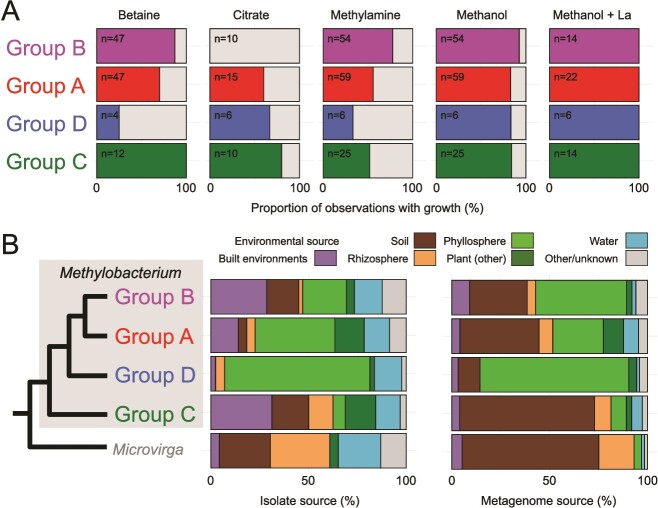
Summary of phenotypic and ecological information available for four *Methylobacterium* groups. (A) Report of *Methylobacterium* group’s ability to use various carbon sources (betaine, citrate, methylamine, and methanol—Both with and without lanthanide). For each group and each carbon source, the percent of observations with detected growth is shown (n = total number of observations; see Table S1 for references). (B) Summary of environmental source for each group. Left: Environmental sources of isolated bacteria. For each group, the information of environmental source of isolation comes from isolates from which genomes were previously analyzed [13]. Proportions were weighted by the number of isolates per species to avoid overrepresentation. Right: Environmental sources of metagenomes [19].

Although the basal group C is distinct by all methods, the genetic boundaries of the later-diverging groups A, B, and D are more difficult to delineate. For instance, deep-branching species from group A, like *Methylobacterium jeotgali*, *Methylobacterium soli*, and relatives, share more ancestry in gene content with groups B and D than with other species from group A (Fig. 2D). Similarly, *M. soli, M. jeotgali, M. trifolii*, *Methylobacterium cerastii*, and relatives share more synteny with some species from group D than with other species from group A (Fig. 2D). Because of these deep-branching species, no clear threshold in ANI could be defined to distinguish groups A, B, and D (Fig. S1). Many of these deep-branching species also showed discrepancies between different individual gene phylogenies explored by Green and Ardley. Together with the inconsistencies of these species between the consensus phylogeny, synteny, gene content and ANI, these observations suggest that groups A, B, and D may have experienced too many gene exchanges since their divergence to be considered as distinct genera [13].

The unfortunate renaming of *Methylobacterium* based on the phylogenetics of the 16S rRNA gene should serve as a wider warning for others in microbial genetics. The 16S rRNA gene is widely used as a marker in prokaryote taxonomy but does not always accurately reflect evolution below the genus level [17] for several reasons: 1) insufficient polymorphism for robust phylogenetic reconstructions; 2) large variation in 16S rRNA gene copy number among groups A (4–6), B (4–5), D (3–4), and C (6–13), sometimes resulting in within-species sequence variation above the inter-species variation; 3) the 16S rRNA gene has its own evolutionary history, which does not reflect the consensus phylogenetic tree accounting for ancestry. This applies to any gene potentially subject to selection or horizontal gene transfers, and with the availability of genomics, one should prioritize inferring taxonomy upon consensus phylogenetic reconstructions from several dozen core genes or from whole-genome information, rather than from a single marker gene.

## Phenotypic evidence against *Methylorubrum*

To further support the split into *Methylorubrum*, Green and Ardley proposed the use of phenotypic data such as the utilization of methylotrophic substrates, growth on multi-carbon compounds, and pigmentation differences. The ability to use methanol as a sole carbon and energy source has been historically used to define *Methylobacterium sensu lato* [18]. This criterion can be retained as all species carry pathways that allow methanol assimilation (Table S1, Fig. 2E), and, so far, there have been no strains unable to use methanol as a sole carbon source, particularly with added lanthanides (Table S1, Fig. 3A). It was suggested that growth on methylamine distinguishes group B (*Methylorubrum*) from the other groups, but this does not serve as a good distinguishing feature, as methylamine use is found throughout groups A, C, and D (Table S1, Figs 2E and3A). The other substrates used by Green and Ardley to distinguish subgroups of *Methylobacterium* were betaine and citrate. Betaine is an *N*-trimethylated glycine that is used by plants as an osmolyte which helps protect against abiotic stress and thus would be an important carbon source for plant-associated bacteria [20]. Citrate is a well-known metal ion chelator in the soil and can be used by a wide variety of bacteria as a carbon source [21]. Although there may be some trends in the available data, such as the lack of citrate utilization in group B strains that have been tested, otherwise both substrates are used by some members of each group throughout the genus (Fig. 3B).

## Distribution of *Methylobacterium* in the environment

Whereas the phenotyping of strains on selected substrates is liable to be arbitrary in terms of which are chosen and have an uncertain connection to their ecology, it is perhaps preferable to use the occurrence in the environment in distinguishing them. By looking at both the isolation source and metagenome location, we can determine a clear differentiation between group C and groups A, B, and D [19]. The main environmental sources of species from group C were soil and rhizosphere, similar to the outgroup *Microvirga* (Fig. 3B). In contrast, the later-diverging groups (A, B, and D) are mostly associated with the phyllosphere. These data suggest that, although all groups can be found across varied environments, there is no evidence that the strains that had been assigned to *Methylorubrum* have a distinct ecology from other *Methylobacterium*.

## Conclusion and future directions

Phylogenomic, phenotypic, and ecological data all provide evidence supporting removing the name *Methylorubrum* [10] and reinstating *Methylobacterium sensu lato* [1]. Accordingly, we call for the definitive adoption of *Methylobacterium* emendation as previously proposed [12]. Although the genus *sensu lato* still contains a large amount of diversity, the naming of *Methylorubrum* did not address this problem. We believe the clearest way to address the large diversity with *Methylobacterium* would be to rename the C group as it follows the consistent pattern of being phylogenetically distinct, phenotypically different, and found in unique locations. Work on this renaming is outside the scope of this perspective and will be handled in proper discourse.

These challenges experienced with *Methylobacterium* highlight the broader issues in bacterial taxonomy, where the resolution of single gene data, especially from 16S rRNA gene sequences, might not provide a clear-cut distinction on the genus level. We hope that this perspective can show how renaming a genus based on single gene phylogeny in the absence of genomic information can fail to capture true relationships among organisms. With the onset of modern genomic techniques, the standard should be to combine information from core genome phylogeny, genome architecture, gene content, and ANI to distinguish genera and to prioritize metagenomic occurrence over arbitrary phenotypes to evaluate whether there is a realized difference in phenotype between taxa.

## Supplementary Material

Figure_S1_wraf011

Table_S1_wraf011

## Data Availability

R codes and related data were deposited on Github (https://github.com/JBLED/Methylobacterium-reinstatement.git).
