# Supplementary material for: Led astray by 16S rRNA: phylogenomics reaffirms the monophyly of Methylobacterium and lack of support for Methylorubrum as a genus
Source: ISME J. 2025 Jan 21;19(1):wraf011. doi: 10.1093/ismejo/wraf011 (PMC11833323; doi:10.1093/ismejo/wraf011)
Supplement: Figure_S1_wraf011 [file figure_s1_wraf011.pdf]

Figure S1: Classification of *Methylobacterium* lineages according to Average Nucleotide Identity (ANI). ANI was estimated with IMG [1] from 213 *Methylobacteriaceae* genomes previously used (Leducq *et al.* 2022) and classified in 23 *Methylobacterium* lineages, *Microvirga* and *Enterovirga*. Lineages are displayed according to the consensus phylogeny from Fig. 2A (top; only topology is displayed). Lineages were grouped together for different ANI thresholds in the range of 79-85% (Table at bottom).

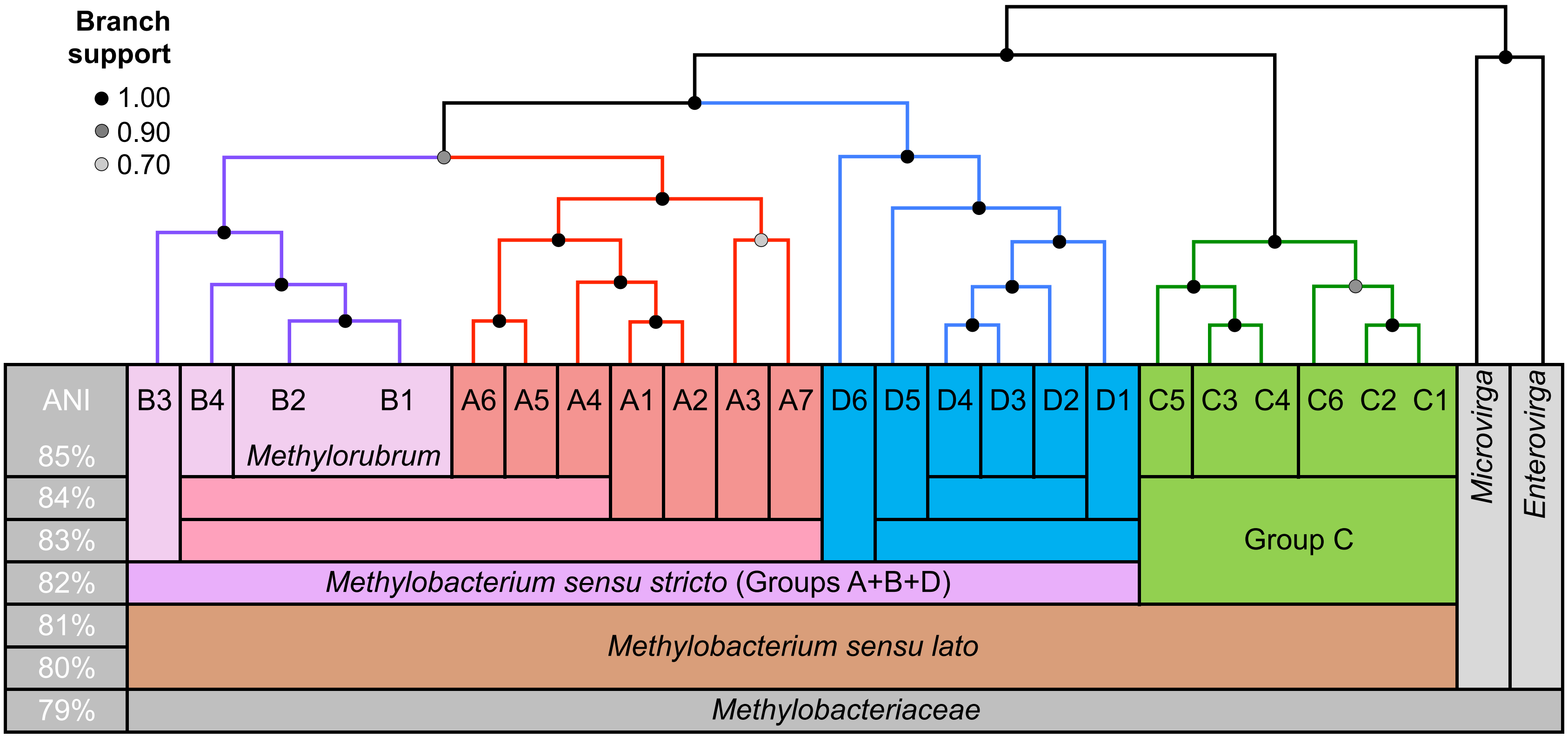

1. Chen I-MA, Chu K, Palaniappan K, Ratner A, Huang J, Huntemann M, et al. The IMG/M data management and analysis system v.7: content updates and new features. *Nucleic Acids Research* 2023; **51**: D723–D732.
